# Midbrain Dopaminergic Neuron Development at the Single Cell Level: *In vivo* and in Stem Cells

**DOI:** 10.3389/fcell.2020.00463

**Published:** 2020-06-25

**Authors:** Emilía Sif Ásgrímsdóttir, Ernest Arenas

**Affiliations:** Division of Molecular Neurobiology, Department of Medical Biochemistry and Biophysics, Karolinska Institutet, Stockholm, Sweden

**Keywords:** dopaminergic neuron, stem cell, progenitor, radial glia, single cell, machine learning, cell replacement, Parkinson’s disease

## Abstract

Parkinson’s disease (PD) is a progressive neurodegenerative disorder that predominantly affects dopaminergic (DA) neurons of the substantia nigra. Current treatment options for PD are symptomatic and typically involve the replacement of DA neurotransmission by DA drugs, which relieve the patients of some of their motor symptoms. However, by the time of diagnosis, patients have already lost about 70% of their substantia nigra DA neurons and these drugs offer only temporary relief. Therefore, cell replacement therapy has garnered much interest as a potential treatment option for PD. Early studies using human fetal tissue for transplantation in PD patients provided proof of principle for cell replacement therapy, but they also highlighted the ethical and practical difficulties associated with using human fetal tissue as a cell source. In recent years, advancements in stem cell research have made human pluripotent stem cells (hPSCs) an attractive source of material for cell replacement therapy. Studies on how DA neurons are specified and differentiated in the developing mouse midbrain have allowed us to recapitulate many of the positional and temporal cues needed to generate DA neurons *in vitro*. However, little is known about the developmental programs that govern human DA neuron development. With the advent of single-cell RNA sequencing (scRNA-seq) and bioinformatics, it has become possible to analyze precious human samples with unprecedented detail and extract valuable high-quality information from large data sets. This technology has allowed the systematic classification of cell types present in the human developing midbrain along with their gene expression patterns. By studying human development in such an unbiased manner, we can begin to elucidate human DA neuron development and determine how much it differs from our knowledge of the rodent brain. Importantly, this molecular description of the function of human cells has become and will increasingly be a reference to define, evaluate, and engineer cell types for PD cell replacement therapy and disease modeling.

## Introduction

Parkinson’s disease (PD) is the second most common neurodegenerative disorder, and its prevalence continues to increase as the population ages (Poewe et al., 2017). Clinically, PD is characterized by a classical triad of motor symptoms: rigidity, tremor, and bradykinesia (Lees et al., 2009). However, patients can also experience a broad spectrum of non-motor symptoms, including cognitive and sensory symptoms (Schapira et al., 2017). The two main pathological features of PD are the progressive loss of midbrain dopaminergic (mDA) neurons in the substantia nigra pars compacta (SNc), and the intraneuronal accumulation of α-synuclein enriched protein aggregates, termed Lewy bodies and Lewy neurites. The loss of mDA neurons in the SNc primarily affects the nigrostriatal pathway, resulting in a nigrostriatal dopamine deficiency, which causes many of the motor symptoms associated with PD (Fahn, 2003).

Current treatment options for PD aim to correct the loss of striatal dopamine through pharmaceutical intervention, either with drugs that modulate DA neurotransmission or that increase dopamine levels in the brain, such as L-DOPA. As an alternative treatment, patients that develop drug-related complications can have electrodes surgically implanted for deep brain stimulation, which compensate for the loss of DA neurotransmission by inhibiting excitatory neurotransmission. However, all of these treatments are symptomatic, and they neither address the underlying cause of the disease nor prevent the progressive degeneration of mDA neurons. Moreover, as the disease progresses, these treatments lose efficacy and lead to undesirable side-effects, further highlighting the need for PD treatment strategies that address the underlying cause of the disease, rather than its symptoms (Brichta et al., 2013).

In addition to novel therapies that address the underlying cause of PD, there is a clear need for regenerative treatments capable of replacing the mDA neurons already lost in PD patients at the time of diagnosis. Therefore, cell replacement therapy is considered a promising treatment option as it could both provide a more physiological delivery of dopamine and replace other important functions of mDA neurons, such as the delivery of trophic factors, which can support other cell types within the basal ganglia that connect with mDA neurons. Human fetal ventral midbrain (VM) tissue has been successfully used for cell transplantation in clinical trials and has provided proof of concept for cell replacement therapy in PD (Arenas, 2010; Lindvall and Björklund, 2011). Notably, the patients who showed the most significant improvement after transplantation were able to discontinue their PD medication and have remained asymptomatic for over 20 years (Kefalopoulou et al., 2014). However, human fetal tissue is difficult to obtain, and differences in tissue quality, how the cells are prepared for transplantation, the surgical procedures employed, and differences in immunosuppression strategies after transplantation have led to variable results. These include a lack of improvement, and, in some cases, undesirable side effects such as graft-induced dyskinesias (Barker et al., 2015). These studies have led to a new clinical trial using human fetal VM tissue (TRANSEURO, NCT01898390), which has been designed to capture all the variables underlying the success of previous clinical trials. TRANSEURO is expected to facilitate the identification of optimal variables for cell transplantation and pave the way for the development of more efficient cell transplantation protocols that may also be applicable to other cell preparations. Indeed, the widespread clinical use of human fetal tissue for transplantation in PD patients is severely limited by several factors including ethical concerns regarding the use of tissue from aborted human embryos, difficulties in standardizing cell composition and quality, as well as the limited availability of this type of tissue (Barker et al., 2015). Due to this, alternative donor sources of mDA neurons for PD cell replacement therapy, such as human pluripotent stem cells, have been explored and developed (Allan et al., 2010; Arenas et al., 2015a; Barker et al., 2015, 2019; Lindvall, 2016; Björklund and Lindvall, 2017; Studer, 2017; Sonntag et al., 2018).

Human pluripotent stem cells (hPSCs) have emerged as an ideal source of material for cell transplantation since they can expand indefinitely and provided the right cues, they can differentiate into any cell type of the body. However, to guide the differentiation of hPSCs into a specific cell type, it is necessary to have a detailed understanding of the developmental steps leading to the generation of that cell type. In the case of PD, mDA neurons are the cell type of interest, and their development has been intensively studied in recent years (Arenas et al., 2015a; Blaess and Ang, 2015; Bodea and Blaess, 2015; Brignani and Pasterkamp, 2017; Smidt, 2017; Brodski et al., 2019; Poulin et al., 2020). However, most of our current knowledge comes from studying mouse mDA neuron development and our knowledge of human midbrain development still remains quite limited.

In mice, mDA neurons arise from the VM region, after regional specification of the neural tube. This regionalization process is accomplished through the combined action of two important signaling centers: the floor plate and the isthmic organizer. In this review, we will briefly discuss these two signaling centers, but for a detailed description, the reader is directed to the many excellent previous reviews in this area, including (Joyner et al., 2000; Martínez, 2001; Wurst et al., 2001; Placzek and Briscoe, 2005; Wilson and Maden, 2005; Nakamura et al., 2008; Cohen et al., 2013).

The floor plate is a specialized glial structure located along the ventral midline of the developing neural tube. This area has classically been considered a structural, non-neurogenic region that acts as a signaling center and contains radial glia cells, which secrete morphogens, such as Sonic hedgehog (Shh), that pattern the neural tube in the dorsoventral axis and specify neural identities (Placzek and Briscoe, 2005). However, fate-mapping studies have shown that unlike other floor plate radial glia, radial glia cells in the midbrain floor plate can undergo neurogenesis to give rise to mDA neurons (Bonilla et al., 2008). The second signaling center, the isthmic organizer (IsO), is located at the midbrain-hindbrain boundary and is established through the coordinated expression and mutual repression of the transcription factor *Otx2* in the midbrain (Millet et al., 1996; Broccoli et al., 1999) and *Gbx2* in the hindbrain (Wassarman et al., 1997; Millet et al., 1999). The IsO secretes the morphogens *Wnt1* on the midbrain side and *Fgf8* on the hindbrain side (Joyner et al., 2000; Puelles et al., 2004), which induces the expression of *Wnt1* in the VM floor plate; a necessary step for the establishment of the midbrain progenitor domain and for mDA neurogenesis (Joyner et al., 2000; Prakash et al., 2006; Andersson et al., 2013).

After specification, mDA progenitors residing in the ventricular zone (VZ) of the floor plate begin to express two transcription factors required for mDA neuron development, *Foxa2* (Ferri et al., 2007) and *Lmx1a* (Andersson et al., 2006b). These progenitors then expand and subsequently undergo neurogenesis, a process regulated by *Neurog2* (Kele et al., 2006) that results in the generation of post-mitotic mDA neuroblasts expressing the transcription factor *Nr4a2* (*Nurr1*) (Zetterström et al., 1997). Following neurogenesis, mDA neuroblasts migrate, first in a radial manner following the processes of the radial glia and subsequently in a tangential manner, toward their final destinations in the mantle zone (MZ) (Kawano et al., 1995). During this migration process, the neuroblasts continue to differentiate and acquire the expression of transcription factors required for mDA neuron development such as *Pbx1* (Villaescusa et al., 2016) and *Pitx3* (Smidt et al., 2004; Maxwell et al., 2005; Veenvliet et al., 2013), as well as genes that identify mDA neurons and are necessary for their function, including the rate-limiting enzyme for dopamine synthesis, tyrosine hydroxylase (*Th*) (Molinoff and Axelrod, 1971), the vesicular monoamine transporter (*Vmat2/Slc18a2*), and the dopamine transporter (*Dat/Slc6a3*) (Miller et al., 1999).

While human mDA neuron development is thought to follow similar principles, little is currently known about the cell type composition of the human VM and the molecular programs that govern human mDA neuron development. Histological analysis of human fetal midbrain tissue has provided some insights into the spatial and temporal organization of the human VM and mDA neuron development. The spatial organization of the human VM appears to correspond principally with the organization of the murine VM, with the three layers of the ventricular, intermediate and mantle zones (VZ, IZ, and MZ) clearly identifiable. Furthermore, human mDA neuron development also appears to follow a similar sequence of events to that of murine mDA neuron development. For instance, the human midbrain floor plate is defined by the expression of *LMX1A* and *FOXA2*, with the *FOXA2* domain extending further laterally into the basal plate (Nelander et al., 2009; Marklund et al., 2014). Moreover, in the VZ of the human floor plate, the pro-neural factor *NEUROG2* overlaps with the expression of *LMX1A*, and in the IZ and MZ, cells expressing key determinants of post-mitotic mDA neurons, *NR4A2*, *PITX3*, and *TH*, can be observed (Nelander et al., 2009).

The information gained from studying mDA neuron development, whether that be in the murine or human VM, has in recent years led to the development of several protocols that direct the differentiation of hPSCs into functional mDA neurons capable of rescuing motor deficits in rodent and non-human primate models of PD (Kriks et al., 2011; Kikuchi et al., 2017; Kirkeby et al., 2017), as well as protocols compliant with good manufacturing practice (GMP) (Doi et al., 2014; Kirkeby et al., 2017; Nolbrant et al., 2017). Last year, following successful preclinical trials in animal models of PD, the first clinical trial using human induced pluripotent stem cells (hiPSCs) as a cell source for transplantation was initiated (Takahashi and Price-Evans, 2019) and other trials using human embryonic stem cells are impending (Barker et al., 2017; Studer, 2017; Sonntag et al., 2018). However, though hPSC-derived mDA neurons have already entered clinical trials, the composition and quality of the cell preparations used or aimed for transplantation have not been fully elucidated at the single-cell level.

With the advent of single-cell RNA sequencing (scRNA-seq), it has become possible to profile the transcriptome of every individual cell in a tissue and to define their molecular signatures in an unbiased and systematic manner. Moreover, analyzing cells at different stages of development by scRNA-seq can provide a description of developmental processes at an unprecedented depth and resolution (Linnarsson and Teichmann, 2016). Recent studies have examined the cellular diversity of mDA neurons at the single cell level in mouse or in human. Microarray or scRNA-seq analysis of postnatal murine mDA neurons (Poulin et al., 2014; La Manno et al., 2016; Hook et al., 2018; Kramer et al., 2018; Saunders et al., 2018; Tiklová et al., 2019a) has led to the identification of 5–7 murine mDA neuron cell types and novel marker genes (reviewed in Poulin et al., 2020). However, the molecular diversity of the adult human brain remains largely to be explored and so far only the two previously known human adult mDA cell types, with *substantia nigra* (SNc) and ventral tegmental area (VTA) phenotypes, have been described (Nichterwitz et al., 2016).

Less is known about the development of embryonic mDA neurons at the single cell level. While Kee et al. (2017) and Hook et al. (2018) detected embryonic mDA neurons in the murine midbrain, no subtypes of embryonic mDA neurons were identified. In contrast, our analysis of murine and human midbrain development unraveled the presence of three embryonic mDA neuron subtypes in both species (La Manno et al., 2016). Moreover, our study provided a first classification of the cell types in the developing murine and human VM, identifying both novel cell types and marker genes; thus, providing new insights into early mDA neuron development and the diversification of the mDA lineage into different embryonic mDA neuron subtypes. Additionally, in the study by La Manno et al. (2016), a systematic comparison of scRNA-seq data of human and murine development was performed, allowing for the comparison of the human and murine VM at the single-cell level. This study provided the first unbiased and systematic classification of the cell types in the developing human midbrain and made it possible to identify differences between human and murine midbrain development.

In the next sections, we focus on the differences between human and murine midbrain development as identified by scRNA-seq. These include differences in cell-type composition, temporal dynamics of development, and the expression of transcription factors at the single-cell level. Furthermore, we describe how the knowledge gained from scRNA-seq analysis can be used to assess the quality of DA neurons generated *in vitro* from hPSCs as well as to guide the improvement of mDA neuron differentiation and reprogramming protocols. We argue that a detailed single-cell level knowledge of the cell preparations being used for cell replacement therapy is necessary to identify the cell types required for functional replacement as well as any unnecessary or undesirable cell types in the preparation. We expect that such knowledge will improve the therapeutic potential and safety of future cell preparations for cell replacement in PD and that the strategy followed here will be useful in addressing the challenge of performing cell replacement in other tissues or organs.

## Human mDA Neuron Development at the Single Cell Level

We recently used scRNA-seq to analyze and compare the human and murine VM at different stages of development, covering mDA neuron specification, neurogenesis and differentiation in both the human (weeks 6–11) and the mouse (E11.5 – E18.5) (La Manno et al., 2016). We used the Fluidigm C1 system, STRT-Seq and Back-SPIN to analyze a total of 1977 VM cells from 10 human embryos and 1907 cells from 271 murine embryos. From this analysis, 25 cell types were identified in the human VM, whereas 26 cell types were identified in the murine VM. The cell types identified in the human and murine VM included early proliferating cells such as radial glia and progenitors; post-mitotic cells, such as neuroblasts and neurons; as well as microglia and cells comprising the vasculature. In the following sections, we describe in greater detail some of the cell types identified in the developing human VM and discuss how these cell types compare to their homologous cell types in the murine VM.

However, it should be noted that while our scRNA-seq analysis provided the first unbiased classification of cell types in the developing human VM, it remains to be determined whether sampling a greater number of cells from additional human embryos will lead to the identification of additional cell types or a better definition of the cell types described in this review. As such, efforts are currently underway to sequence a greater number of cells and embryos, at additional developmental stages, using new and improved scRNA-seq methods, which are expected to improve the resolution of the analysis described here.

### Human VM Radial Glia

Our analysis of the developing human VM (weeks 6–11) identified a greater diversity of radial glia cell types compared to the mouse (embryonic days 11.5–18.5) (La Manno et al., 2016) and to what has previously been described (Ostrem et al., 2017). While three distinct types of radial glia cells (Rgl1-3) were detected in the mouse VM, five molecularly defined radial glia cell types (Rgl1, 2a-c, 3) were found in the human VM by scRNA-seq. However, single-molecule fluorescent *in situ* hybridization (smFISH) analysis also revealed the presence of Rgl2c in the embryonic mouse VM, suggesting that other homologous human Rgl2 subtypes may also be present in the mouse VM. Further experiments will be necessary to determine whether equivalent midbrain Rgl cell types exist in both species and whether their spatial organization is similar.

**FIGURE 1:**
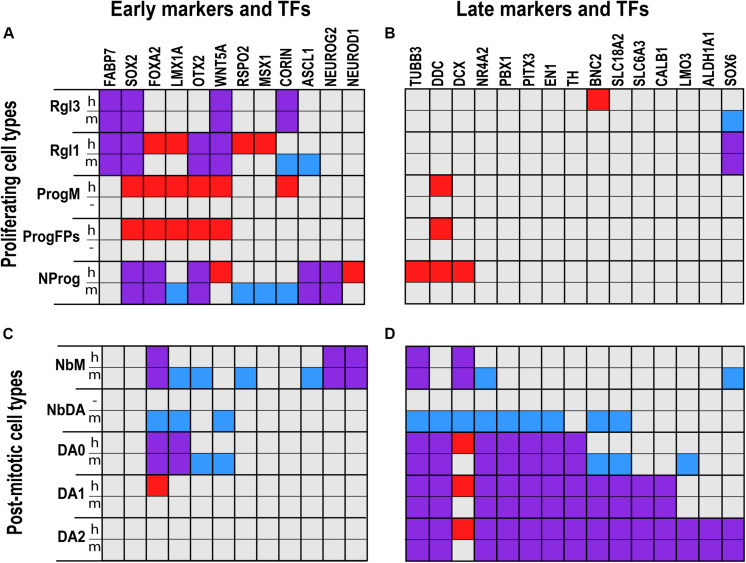
Comparative analysis of gene expression in defined cell types of the developing human and murine ventral midbrain as assessed by scRNA-seq. Proliferating **(A,B)** and post-mitotic cell types **(C,D)** expressed distinct early **(A,C)** and late **(B,D)** marker genes and transcription factors (TFs). Squares in red denote genes detected only in human; in blue, detected only in mouse; and in purple, detected in both human and mouse. Gene expression was more similar across species in radial glia (Rgl1, 3) and dopaminergic neurons (DA0-2) than in neuronal progenitors (NProg) or neuroblast medial (NbM). Cells such as medial progenitors (ProgM) or lateral and medial floorplate progenitors (ProgFPs) were only found in human, whereas the dopaminergic neuroblast (NbDA) was only found in mouse. Cell type and gene expression from La Manno et al. (2016).

Homologous radial glia cell types in both species were found to share the expression of certain genes, such as the neural stem/progenitor transcription factor (TF) *Sox2* (Graham et al., 2003), and the expression of a fatty acid binding protein induced by Notch signaling (*Fabp7*) (Anthony et al., 2005) (Figure 1A). However, each radial glia cell type was found to have a distinctive expression profile, suggesting that they may serve separate functions during VM development. For instance, Rgl1, a cell type present in both the floor plate and the basal plate of the VZ during mouse VM development, is characterized by the expression of TFs implicated in neurogenesis and mDA neuron specification. Human and mouse Rgl1 share the expression of the pro-neural basic helix-loop-helix (bHLH) factor *Ascl1*, as well as the TF *Otx2*, both of which have many roles in mDA neuron development, from specification to neurogenesis (Figure 1A) (Vernay et al., 2005; Kele et al., 2006; Prakash et al., 2006; Omodei et al., 2008). Additionally, human Rgl1 express other essential determinants of mDA neurons such as the TFs *LMX1A* and *MSX1*, which are required for the specification and differentiation of mDA neurons (Andersson et al., 2006b). Surprisingly, these TFs are not expressed in mouse Rgl1, but rather in a neuronal progenitor cell type present in the mouse VM (see below), suggesting that non-homologous cell types may serve the same function. Thus, while homologous Rgl1 may play a common role in specification and neurogenesis, their role in mDA neuron development may differ.

The second radial glia cell type, Rgl2, is a cell type that in mice is found exclusively in the basal plate, first in the VZ and then in the MZ, suggesting that Rgl2 migrate away from the VZ during VM development. Human and mouse Rgl2 share the expression of several gliogenic markers, such as the glia specific glutamate transporter *Slc1a3* (Torp et al., 1994; Lehre et al., 1995) and the astrocyte markers *Aldoc* (Walther et al., 1998) and *Tnc* (Karus et al., 2011), suggesting that Rgl2 cells in both species may play a role in gliogenesis. Interestingly, three molecularly defined Rgl2 subtypes (Rgl2a-c) were identified in the human VM based on their expression profile, indicating that they may serve some more specialized functions in human midbrain development. It is interesting to note that the transcriptional profile of Rgl2c was found to be similar to that of oligodendrocyte precursor cells (OPCs), with these two cell types sharing the expression of *Olig2*, an important OPC marker (Fu et al., 2002). Since Rgl2c is detected at human embryonic week 10 and OPCs are detected around weeks 10–11, we suggest that Rgl2c may be the cell type giving rise to OPCs. Lineage-tracing experiments should be performed in the future to determine whether there is any lineage relation between these two cell types.

The third radial glia cell type, Rgl3, is found predominantly in the mouse floor plate. Both mouse and human Rgl3 are characterized by the expression of several secreted factors implicated in different aspects of midbrain development. These include *Slit1*, *Slit2*, and *Ntn1*, involved in neuronal migration and axon guidance (Livesey and Hunt, 1997; Lin et al., 2005), *Spon1* (Klar et al., 1992), as well as morphogens and growth factors of the transforming growth factor-beta (TGFB) family (Poulsen et al., 1994; Arenas et al., 2015b).

These data suggest that during weeks 6–11 of human embryonic development, Rgl3 may serve the classical role of floor plate radial glia as a signaling center, while Rgl1 could play a role as neurogenic radial glia and Rgl2 in the basal plate could be specialized in gliogenesis. Future studies should focus on addressing the current gaps in our knowledge regarding the role of radial glia in human development. These should include determining the position that human radial glia occupy along the ventral-dorsal and anterior-posterior axis of the VM VZ, and whether radial glia cells can migrate to the IZ and MZ, as suggested by smFISH analysis of the mouse VM (La Manno et al., 2016). Finally, the precise function and contribution of each radial glia cell type to human mDA neuron development remains to be determined, e.g., through experimental ablation of these cell types.

### Progenitor Cell Types in the Developing Human Midbrain

scRNA-seq analysis of the developing human midbrain also revealed a great diversity of progenitor cell types (La Manno et al., 2016), with molecular identities that largely correspond to previously described progenitor domains in the mouse and human VM (Andersson et al., 2006b; Prakash et al., 2006; Ono et al., 2007; Hebsgaard et al., 2009). The human VZ was found to contain five distinct progenitor cell types, which were named based on their molecular identity as well as their presumed location and/or function in the VZ (ProgM [midline progenitor]; ProgFPL [lateral floor plate progenitor], ProgFPM [medial floor plate progenitor]; ProgBP [basal plate progenitor]; NProg [neuronal progenitor]) (La Manno et al., 2016). However, it should be noted that these human progenitor cell types were named after the similarity of their transcriptomes to that of their homologous mouse cell types, but not because their position in the human VM, which still remains to be determined. It is thus possible that some of the human progenitor cell types may occupy a different or unsuspected position in the human VM. A systematic histological identification of the position of human progenitor cell types in the developing human VM will be necessary to define their spatial organization.

Human progenitor cells were found to share some characteristics with radial glia cells, such as the expression of the neural stem cell marker *SOX2* (Graham et al., 2003), and the expression of *RFX4*, a TF that modulates Shh signaling (Ashique et al., 2009). However, they also have specific characteristics that set them apart from radial glia cells, e.g., they all express the chromatin remodeling proteins HMGA1 and HMGB2 (Hock et al., 2007). Additionally, each progenitor is characterized by a unique molecular signature, discussed below.

The midline progenitor (ProgM) is defined by the expression of *CORIN* (Figure 1A), a natriuretic peptide-converting enzyme that marks the murine VM midline (Ono et al., 2007), as well as *TOX*, a transcriptional regulator of *SOX2* (Artegiani et al., 2015), and *JADE-1*, an inhibitor of the Wnt signaling pathway (Chitalia et al., 2008). Both floor plate progenitors (ProgFPL and ProgFPM) are characterized by the expression of *LMX1A*, a transcription factor that specifies mDA neurons and labels the human floor plate (Figure 1A) (Andersson et al., 2006b; Hebsgaard et al., 2009; Nelander et al., 2009). Additionally, the lateral floor plate progenitor (ProgFPL) is identified by a high expression of the morphogen *WNT1*, which is required for midbrain and mDA neuron development in mice (McMahon and Bradley, 1990; Thomas and Capecchi, 1990; Danielian and McMahon, 1996; Prakash et al., 2006; Andersson et al., 2013; Yang et al., 2013), and *ZEB2*, a transcription factor that regulates mDA progenitor proliferation and neurogenesis (Yang et al., 2018). Conversely, the medial floor plate progenitor (ProgFPM) is characterized by low *WNT1* expression. Basal plate progenitors (ProgBP) were identified by the expression of *FOXA2* in the absence of the floor plate markers mentioned above, such as *WNT1* and *LMX1A*. The final progenitor identified in the human VZ, NProg, is not characterized by the expression of genes in a defined medial-lateral compartment, but rather by the expression of pro-neural genes such as *NEUROG2* and *ASCL1* (Andersson et al., 2006a; Kele et al., 2006); genes known to regulate mDA neurogenesis such as *OTX2* (Omodei et al., 2008) (Figure 1A), and early neuronal markers such as *TUBB3* (Figure 1B). We thus hypothesize that this human progenitor reflects a class of neurogenic progenitors capable of giving rise to post-mitotic neuroblasts and neurons. A similar NProg was also identified in the mouse developing midbrain. In addition to *Neurog2* and *Ascl1*, the mouse NProg also express genes found in the human ProgM and ProgFPs, such as *Corin* and *Lmx1a*, respectively (Figure 1A). This observation and the fact that proliferative progenitors with distinct mediolateral identities were not identified in the mouse suggests that mouse progenitors identified at E11.5 are in a more differentiated neurogenic state compared to human week 6. Further analyses will be needed to determine whether the progenitor diversity found in the human VM can be identified at earlier stages in mice and whether such diversity may also lead to further subdivisions of the NProg cluster if ProgBP, ProgFPL, ProgFPM, or ProgM progenitors retain their compartment identity while undergoing neurogenesis.

### Human VM Post-mitotic Cells

In addition to the variety of progenitor cell types detected in the VZ, our scRNA-seq analysis also revealed a great diversity of post-mitotic cell types in the developing human and murine VM. Post-mitotic cells in the developing VM were commonly characterized by a loss of proliferative markers and the gain of markers such as *TUBB3* (β-III-tubulin), an early cytoskeletal neuronal marker (Jiang and Oblinger, 1992), and *SNAP25*, a late synaptic marker (Söllner et al., 1993). Post-mitotic cells can be further divided into neuroblasts and neurons. Neuroblasts are immature migratory cells born from progenitor cells that progressively mature and differentiate into neurons, the cell types expressing all the machinery required for the synthesis, release, and uptake of neurotransmitters. While all neuroblast cell types share some common features, they can be distinguished by the differential expression of transcription factors that define their lineage and spatial position. Comparison of mouse and human single-cell transcriptomes revealed the presence of three common neuroblast cell types in the mouse and human. These cells were named based on their presumed location in the developing mouse VM (NbM [neuroblast medial], NbML1 and 5 [neuroblast medio-lateral]), with NbM and NbML1 being the earliest appearing and the most abundant neuroblasts in the developing VM of both species (La Manno et al., 2016).

In both human and mouse, NbM was characterized by the expression of several bHLH factors such as *NEUROD1* and *NEUROG2* (Figure 1C), which play essential roles in neurogenesis and differentiation (Kele et al., 2006; Dennis et al., 2019). Additionally, mouse NbM was found to express *Nr4a2* (*Nurr1*) (Figure 1D), a nuclear receptor required for mDA neuron development (Zetterström et al., 1997). While expression levels of *NR4A2* were lower in human NbM, histological analysis of the developing human VM has shown that *NR4A2* is present in tyrosine hydroxylase-negative (*TH*^–^) neuroblasts in the floor plate and *TH*^+^ mDA neurons in the VM (Marklund et al., 2014). The same expression pattern of *Nr4a2* can also be seen in histological analysis of the mouse developing midbrain (Villaescusa et al., 2016), indicating that in both the human and mouse VM, NbM differentiate into mDA neurons. Additionally, *Nr4a2* was also expressed in a more differentiated mouse mDA neuroblast (NbDA) (Figure 1D), a cell type which was identified by scRNA-seq in mouse but not in the human VM (La Manno et al., 2016).

The second most abundant neuroblast in human and mouse is the mediolateral neuroblast 1 (NbML1), which is characterized by the expression of the transcription factor *Nkx6-2* (La Manno et al., 2016). In the mouse, NbML1 cells are defined by the expression of *Nkx6-2* and *Cartpt* (cocaine- and amphetamine-regulated transcript prepropeptide), two markers labeling in the IZ/MZ of the basal plate (La Manno et al., 2016). Cart is transiently expressed in an embryonic population in the MZ of the presumptive dorsal tegmentum, as well as in the embryonic and adult Edinger–Westphal Nucleus in the rat (Brischoux et al., 2002). In the mouse, *Cartpt* is expressed by several postnatal neurons in the dorsal-medial tegmentum, including the Edinger–Westphal nucleus, periaqueductal gray, oculomotor and trochlear neurons (Allen brain atlas, 2004, image 18). In addition, 15% of the red nucleus (RN) neurons in mice express *Nkx6-2* (Prakash et al., 2009; Moreno-Bravo et al., 2010). We thus suggest that NbML1 cells may give rise to different *Cartpt*+ or *Nkx6-2*+ neuron types that will occupy the dorsal-medial tegmentum. Ultimately, spatial transcriptomics and lineage tracing experiments will be, respectively, required to determine the position and cell types generated by NbML1 in mouse and human.

Besides neuroblasts, several homologous neuronal cell types were detected in the developing human and murine VM, including dopaminergic; serotonergic; GABAergic; oculomotor and trochlear nucleus (OMTN) and RN neurons. All the neuronal cell types were identified based on the expression of components necessary for neurotransmission. This included the rate-limiting enzyme for neurotransmitter synthesis, the vesicular transporter responsible for loading the neurotransmitter into synaptic vesicles as well as the neurotransmitter reuptake transporters. In the case of mDA neurons, they were characterized by the expression of *TH*, the rate-limiting enzyme in dopamine synthesis, *SLC18A2* (*VMAT2*), and *SLC6A3* (*DAT*). However, three distinct types of embryonic mDA neurons could be detected in the developing human and murine VM (DA0-2) (Figure 1D).

The earliest embryonic mDA neuron, DA0, was detected at week 6 (E11.5 in mouse). This cell type expresses *TH* but lacks *SLC6A3*, and thus it was classified as an incomplete DA neuron not yet capable of neurotransmission. At week 7 (E12.5 in mouse), the first mDA neuron cell type (DA1) that expresses *SLC6A3*, thus fulfilling all the criteria for neurotransmission, was detected. A second mDA neuron (DA2) was detected at week 8 (E13.5 in mouse) and was found to additionally express *SOX6*, a transcription factor (Panman et al., 2014), *LMO3*, a transcriptional co-activator of *Pitx3* (Bifsha et al., 2017) and aldehyde dehydrogenase 1 family member A1 (*ALDH1A1*), an enzyme involved in DA catabolism (Goldstein et al., 2013) as well as the synthesis of retinoic acid (McCaffery and Drager, 1994) and GABA (Kim et al., 2015). In the adult murine midbrain, the expression of these three genes overlap only in the ventral tier of the SNc pars compacta. Indeed, mDA neurons expressing *Aldh1a1* are located in the ventral part of the ventral tegmental area (VTA), and the ventral tier of the SNc pars compacta (Galter et al., 2003; Poulin et al., 2018). *Sox6* expressing mDA neurons are found in both the ventral and dorsal tier of the SNc and the dorsolateral VTA (Panman et al., 2014; Poulin et al., 2018). *Lmo3* is preferentially expressed in SNc mDA neurons (Bifsha et al., 2017). These results suggest that embryonic mouse and human DA2 neurons are likely candidates to give rise to mDA neurons of the SNc. However, the molecular diversity of the adult human midbrain and postnatal mDA neurons remains to be elucidated at the single-cell level. Future studies should aim to characterize this diversity and to understand the lineage relationship between embryonic DA neurons and postnatal/adult mDA neuron subtypes.

## Temporal Differences Between Homologous Cell Types in Human and Mouse Development

To determine potential differences in temporal dynamics between human and mouse midbrain development, investigators have traditionally relied on histological analysis and examined specific markers that define cell types at different developmental time-points. For instance, histological analysis of the human embryonic midbrain has revealed that human mDA neurons, identified by *TH* expression, appear at weeks 5–6 post-conception, mDA neurogenesis peaks around w7–8 and then ceases by w10–11 (Freeman et al., 1991; Almqvist et al., 1996; Puelles and Verney, 1998; Verney et al., 2001), resulting in ∼400.000–600.000 human mDA neurons (German et al., 1983; Pakkenberg et al., 1991). In contrast, murine mDA neurogenesis takes place in only 6 days, with neurogenesis beginning at E9.5, peaking at E11.5 and then ceasing at around E14.5–15.5 (Bayer et al., 1995), resulting in ∼30.000 murine mDA neurons (Nelson et al., 1996). The significant increase in gestation time for humans would be expected to result in an even greater number of mDA neurons. However, cell cycle length is longer in human progenitors, resulting in neurogenesis not being directly proportional to the increase in gestation time (Kornack and Rakic, 1998). Indeed, it has been estimated that human progenitors in the VZ are only about half as proliferative as those in the mouse (La Manno et al., 2016).

However, to compare developmental time across species in a more precise manner, it is important to consider not one, but a series of neurodevelopmental events. To this end, Workman et al. (2013) developed a generalized linear regression model, which correlates brain development across different species based on a series of anatomical and developmental milestones. This model will be used here to compare the temporal axis of homologous cell types in the developing VM to determine whether differences in temporal dynamics exist between human and mouse midbrain development.

**FIGURE 2:**
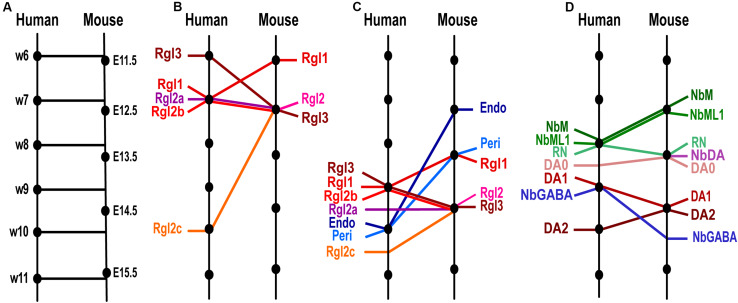
Temporal differences between homologous cell types in human and murine ventral midbrain development as detected by scRNA-seq. **(A)** Model of the time equivalence between human and murine midbrain development, as defined by Workman et al. (2013) based on developmental features. Homologous time-points are indicated with lines perpendicular to the time-line. **(B)** Comparison of the time of appearance of human and mouse Radial glia (Rgl) cell types, showing significant species differences (oblique lines) in Rgl3, Rgl1 and Rgl2c. **(C)** Progenitor and non-neuronal cell types ordered by average time of appearance as detected by scRNA-seq. Note that vascular cells, endothelial (Endo) and pericytes (Peri) appear earlier in mouse than in human and show greater temporal differences than Rgl types. **(D)** Post-mitotic cell types ordered by average time of appearance showing greater temporal species differences in neuroblasts than in neurons. Cell type and time-line from La Manno et al. (2016).

Based on the model by Workman et al. (2013), the human embryonic midbrain at w6 is homologous to mouse E11.5. After that, one week of human midbrain development is less than one embryonic day in the mouse, with w11 being homologous to E15.5 (Figure 2A). However, it is important to keep in mind that while such models allow for comparison between human and mouse midbrain development, it is unclear whether there is a perfect temporal correlation between homologous cell types across the two species. Indeed, the time at which a human or murine homologous midbrain cell type is first detected by scRNA-seq, or the time at which 50% of a certain cell type has appeared (average time of appearance) varies considerably (La Manno et al., 2016) even if equivalent periods of time in human (w6–w11) and mouse (E11.5–15.5) development (according to Workman) are considered.

Temporal differences between mouse and human VM development can already be detected in proliferating cells at early developmental stages, as illustrated by the different order of appearance of VM radial glia cell types. Murine Rgl1 cells are detected at ≤E11.5, while mRgl2 and mRgl3 emerge at E12.5, as assessed by scRNA-seq and smFISH. Conversely, in the developing human midbrain, the earliest radial glia cell type detected by scRNA-seq is hRgl3, appearing at w6 [homologous mouse time (hmt) of E11.5], a day earlier than mRgl3 (E12.5). On the other hand, hRgl1 appears at w7 (hmt ≈ E12.5), a day later than mRgl1 (E11.5). Finally, human Rgl2a-b appear at w7 (hmt ≈ E12.5, a similar developmental stage to the mouse), while hRgl2c is only found at w10 (hmt ≈ E15, i.e., 2.5 days after mRgl2 is detected) (Figure 2B).

Similar species differences are also detected when considering the average time of appearance for each radial glia, although they are less clear (Figure 2C). In human, Rgl1 is found on average slightly later than mouse Rgl1 (w9, hmt ≈ E14 vs. E13.5), while human Rgl3 is found slightly earlier (w9, hmt ≈ E14) than mouse Rgl3 (E14.5). However, human Rgl2 are found at similar stages as mRgl2 (E14.5), with hRgl2b found at w9 (hmt ≈ E14), hRgl2a at w9.5 (hmt ≈ E14.5), and hRgl2c at w10.5 (hmt ≈ E15).

These results, as well as species-specific differences identified in the transcriptional profile of the different Rgl cells, discussed below, raise the interesting question of whether human and murine Rgl cell types serve entirely homologous or some species-specific functions in midbrain development. Gain and loss of function experiments will be required to determine their individual function and how changes in their temporal order of appearance could affect midbrain development. An additional critical issue that also remains to be addressed is the lineage relationship between distinct radial glia subtypes and their relationship to the diverse progenitor cell types in the midbrain.

Temporal differences between human and mouse midbrain development were also found in post-mitotic cell types. For instance, the two most abundant midbrain neuroblast cell types, the medial neuroblast (NbM) and the mediolateral neuroblast type 1 (NbML1), are both found at later stages in human development compared to the mouse. Indeed, the average time at which both cell types are detected in the mouse is E12.5, while their human counterparts are detected on average at w8 (hmt ≈ E13.5, i.e., 1 day later). Other neuroblasts that appear later in development, such as NbGaba, are also detected at non-homologous developmental times, but in this case, they are detected on average at earlier stages in human (w9, hmt ≈ E14), compared to mouse (E15) (Figure 2D). These results indicate that homologous neuroblasts can be found at non-homologous time-points, suggesting that each neuroblast has a unique developmental timeline in each species.

## Differences in the Dopaminergic Lineage and the Time Sequence at Which Cell Types Emerge During Human and Mouse Development

Lineage tracing studies in the mouse have demonstrated that mDA neurons are derived from progenitors that express morphogens such as *Shh* and *Wnt1* (Zervas et al., 2004; Joksimovic et al., 2009; Blaess et al., 2011; Brown et al., 2011), as well as the glutamate astrocyte transporter *Slc1a3* (*Glast*) (Bonilla et al., 2008) and the floor plate marker *Corin* (Ono et al., 2007). *Shh* expression in the VM floor plate is dynamic and has been shown to delineate different progenitor domains, including progenitor domains that will later give rise to DA, RN and OMTN neurons. All mDA neurons appear to arise from VM floor plate progenitors that express *Shh* and respond to high levels of *Shh* signaling (as assessed by *Gli1* expression), with mDA progenitors expressing *Shh* between E8.5–11.5 and responding to Shh signaling between E7.5–E9.5. *Shh* expressing progenitors contribute the most significant amount of mDA neurons at E9.5, at the time when *Shh* expression is detected in the entire *Lmx1a* domain of the FP (Joksimovic et al., 2009; Blaess et al., 2011). This indicates that mDA neurons are derived from progenitors that co-express *Shh* and *Lmx1a*. Conversely, at E11.5, when *Shh* is downregulated in the floor plate midline, *Shh* expressing progenitors no longer contribute mDA neurons (Joksimovic et al., 2009; Blaess et al., 2011). Additionally, fate-mapping studies show that progenitors in the midbrain-hindbrain boundary and VM floor plate expressing *Wnt1* between E7.5-13-5 also generate mDA neurons (Zervas et al., 2004; Prakash et al., 2006; Brown et al., 2011), particularly at E9.5 (Brown et al., 2011). Combined, all the results described above indicate that progenitors in the VM hindbrain boundary and the midbrain floor plate give rise to mDA neurons.

These fate-mapping studies were done at an early developmental time point, making them difficult to interpret in the context of our scRNA-seq data. However, mDA neurons have also been fate mapped from radial glia cells at E10.5–11.5 using the glutamate astrocyte transporter *Slc1a3* (Bonilla et al., 2008), which is expressed in all radial glia cells in the murine VM (mRgl1-3) (La Manno et al., 2016). Furthermore, mDA neurons can be derived from VM progenitors sorted at E13.5 for the midline marker *Corin* (Ono et al., 2007), which is expressed in mRgl1/3 and NProg (La Manno et al., 2016). Thus, comparing scRNA-seq data with previous lineage tracing and FACS sorting suggests that mDA neurons may be derived from mRgl1. However, *Lmx1a*, a gene required for mDA neuron specification (Andersson et al., 2006b), is not expressed in mRgl1, but rather in mNProg and downstream cells in the mDA lineage: mNbM, mNbDA and mDA0 (Figure 1). Additionally, our scRNA-seq data shows that mRgl1, as well as mNProg and mNbM, express *Ascl1* (marking the onset of neurogenesis), mNProg and mNbM express *Neurog2* (a gene required for mDA neurogenesis), while mNbM express *NeuroD1* (a gene downstream in neurogenesis). This suggests a sequence of events in which mRgl1 becomes mNProg, which then gives rise to the first post-mitotic cell, mNbM. Finally, histological and knockout analysis in mice suggest that *Neurog2*+ cells in the VZ (mNProg) give rise to *Nr4a2*+ cells in the IZ (mNbM) (Kele et al., 2006), which in turn subsequently differentiate into *Pbx1*+ mNbDA and Th+ DA neurons (Villaescusa et al., 2016), with the embryonic DA neurons appearing sequentially, from mDA0 to mDA2 (La Manno et al., 2016).

Much less is known about the lineages in the developing human midbrain. However, recent studies in which human embryonic stem cells (hESCs) have been FACS sorted during mDA neuron differentiation have provided some interesting information. Antibodies targeting proteins such as CORIN (Doi et al., 2014), ALCAM (Bye et al., 2015) or CD47 (IAP) (Lehnen et al., 2017), all of which are expressed by hRgl3 and hProgM (La Manno et al., 2016), have been used to isolate progenitors capable of giving rise to human mDA neuroblasts (hNbM) and mDA neurons (hDA0-2). Similarly, *LMX1A*, a gene expressed in hRgl1, hProgM, hProgFPM, and hProgFPL (La Manno et al., 2016), has been used to generate hESC reporter lines that, after sorting, can generate human mDA neuroblasts and neurons (Samata et al., 2016; Niclis et al., 2017). These results suggest that in humans, the first cell type capable of mDA neurogenesis is hProgM at week 6 (instead of mRgl1 in mice). As in the mouse, the next candidate cell type in the DA lineage is hNProg, which expresses several genes involved in mDA neurogenesis (*Ascl1*, *Neurog2*, and *NeuroD1*), followed by hNbM, which expresses *Neurog2* and *NeuroD1*. However, no human NbDA was detected and the next cell type detected in the mDA lineage is hDA0, followed by hDA1 and then hDA2 (Figure 2D), which progressively acquire the expression of mDA neuron subtype markers (Figure 1D).

The results above suggest important species differences in mDA neuron development, such as the first candidate cell type in the human mDA lineage being a non-homologous cell type (hProgM vs. mRgl1), that NbDA is either not found or is a very transient population in humans (see discussion below), and that gene expression in any given cell type of the human mDA lineage is not identical to that in the homologous mouse cell type (Figure 1A). Ultimately, deeper and more extensive scRNA-seq and spatial transcriptomics analysis will improve the definition of these cell types, their expression profile and their positions within the developing midbrain. Moreover, lineage tracing experiments using molecular barcodes and single-cell sequencing methods in human tissue or stem cell preparations (2D or 3D) will be needed to decipher the actual lineage relationship within the mDA lineage and define the kinetics of cell-to-cell transitions during human development.

## Temporal Differences in Lineage Transitions Between Human and Mouse Development

Using the model developed by Workman et al. (2013), the time at which each cell type appears according to the scRNA-seq analysis and the limited lineage information available, it is also possible to estimate how long it takes for a given post-mitotic neuroblast to generate a downstream neuron type. In this way, we find that mouse NbM cells take 2 days to generate DA1 neurons (mNbM found at E12.5 and mDA1 at E14.5), whereas human NbM cells generate DA1 in less than 1 week (w8–8.75), corresponding to less than a day in homologous mouse time (hmt) (Figure 2D). The short time it takes for hNbM cells to generate hDA1 neurons suggests that intermediate cell types in the DA lineage may only exist for a very brief period of time, making them difficult to detect. In support of this hypothesis, we reported that NbDA, a cell type directly downstream of NbM in the mouse (Villaescusa et al., 2016), was not detected in the human midbrain, as it may exist only for a very brief time in human development (La Manno et al., 2016). Further scRNA-seq analysis of the developing human VM will be needed to determine whether this is the case.

Notably, the species difference in the kinetics for generating hDA1 neurons seems to be cell-type specific because other cell populations do not follow the same dynamics. For instance, NbML1 neuroblasts, which are thought to give rise to 15% of red nucleus (RN) neurons, take comparable homologous developmental time in both species; less than a day in the mouse and within half a week in the human (Figure 2D). Thus, currently available data indicate that VM neuroblasts generate neurons following both cell type- and species-specific temporal kinetics.

In addition to neural cells, the cells that make up the vasculature in the midbrain, such as pericytes and endothelial cells, appear relatively late in human development compared to the mouse. While human endothelial cells and pericytes are detected on average at w10 (hmt ≈ E15), they are detected significantly earlier in the mouse, at E13 and E14, respectively (Figure 2C). This finding is somewhat surprising, as a slower vascularization of the human VM would limit oxygen and nutrient supply compared to the mouse midbrain. These conditions may favor the maintenance of cells with anaerobic metabolism such as progenitor cell types and slow down the maturation of neurons in the human VM.

In sum, from this analysis, it becomes apparent that while some cell types are generated at homologous developmental time points, following the model by Workman et al. (2013), others are not. These results suggest that cells do not follow a universal homologous linear timeline across species, but instead follow their own cell type- and species-specific developmental time. This finding may have important implications as cells may become functional at different developmental times, resulting in species-specific cell type assemblies and interactions at the tissue level. Future experiments should aim to elucidate how these observed temporal differences in cell types across species may impact the regulation of gene expression, cell signaling, and metabolism within the tissue. Furthermore, it remains to be determined how such temporal differences may impact key developmental events such as lineage progression, migration, and functional maturation in a species-specific manner.

## Differences in Transcription Factor Expression Between Human and Mouse Cell Types During Midbrain Development

The function of transcription factors in mDA neuron development has been extensively studied in the mouse (reviewed in Arenas et al., 2015a; Blaess and Ang, 2015; Bodea and Blaess, 2015; Smidt, 2017). However, their function in human mDA neuron development remains largely unknown. Our scRNA-seq analysis and comparison of the developing human and murine VM demonstrated that TFs required for mDA neuron development in mice are generally expressed in homologous mouse and human cell types (La Manno et al., 2016). These results suggest that most TFs will form similar transcriptional networks and may serve similar functions in homologous cell types in both species. Nonetheless, key TFs commonly used to identify cells in the mDA lineage are also expressed in non-homologous cell types. In previous sections, we described how homologous cell types in human and mouse differ in gene expression profiles. Here, we examine how the choice of common TFs to identify cell types may impact the identification of cell types both within one species and when comparing across species. In interpreting this data, it should be noted that TFs are usually expressed at low levels and are thus difficult to detect, which may lead to false negative results. It should also be noted that the developmental time at which cells are sampled and the number of cells sampled may have influenced the results to some extent. It thus remains to be determined whether by analyzing additional developmental stages and a greater number of cells with improved sequencing methods, it may be possible to detect additional cell types and differences in gene expression in a more reliable manner. Undoubtedly, as the methodology and data analysis improve, we can in the near future expect to gain a better definition of the cell types and genes that control mDA neuron development.

### Factors Specifying mDA Progenitors

As discussed above, disparities in TF expression between homologous murine and human cell types are already apparent at early developmental stages. Notably, differences in TF expression, such as *LMX1A* in hRgl1 or mNProg, are not isolated events, but rather reflect more profound changes in TF expression signatures, indicating that non-homologous cell types share surprisingly large TF expression signatures. For instance, in the developing human midbrain, hRgl1 expresses several of the TFs that identify mDA progenitors, such as *SOX2*, *OTX2* (Vernay et al., 2005), *FOXA2* (Ferri et al., 2007), *LMX1A*, and *MSX1* (Andersson et al., 2006b). However, in the mouse, the combination of these factors is not expressed in mRgl1, but rather in a non-homologous cell type: the neuronal progenitor (mNProg) (Figure 1A). This observation is both interesting and unexpected because several of these TFs are known to regulate each other and are part of a developmental pathway that controls mDA progenitor identity and the generation of mDA neurons. However, these two cell types, in keeping with their identification as non-homologous cell types, differ in the expression of other TFs. For instance, hRgl1 expresses *LHX3*, which is involved in motor neuron specification (Sharma et al., 1998), while mNProg expresses TFs controlling mDA neurogenesis, such as *Ascl1* and *Neurog2* (Kele et al., 2006). Thus, the expression of these additional TFs may lead to the formation of distinct transcriptional networks, expression profiles, and perhaps even additional functions such as motor neuron development in the case of hRgl1.

Similar observations can be made when different cell types from the same species are surveyed with a combination of markers. For instance, *OTX2*, *FOXA2*, *LMX1A*, and WNT5A are commonly expressed in distinct human proliferating cell types such as hRgl1, hProgM hProgFPM, and hProgFPL (La Manno et al., 2016), which are distinguished from one another by the expression of additional TFs and morphogens that may confer different functions. Thus, commonly used combinations of genes currently thought to identify one cell type can, in fact, identify either multiple cell types within one species or even non-homologous cell types across species. These results emphasize the importance of examining broad marker panels supported by single-cell data or the use of single-cell transcriptomic profiling to identify the correct cell type.

Another interesting finding is that markers such as *OTX2*, *FOXA2*, *LMX1A*, and *WNT5A*, which are used to define generic mDA neuron progenitors, are expressed at significant levels in most early human proliferating cell types (hRgl1, hProgM, hProgFPM, and hPRogFPL). Notably, their expression then rapidly decreases in hNProg and is low during differentiation in human cell types, but not in the mouse. Indeed, unlike mNProg, hNProg expresses significant levels of only two of these four factors: *FOXA2* and *WNT5A* (Figure 1A). Moreover, no human post-mitotic cell type expresses significant levels of more than two of these factors, while several mouse post-mitotic cell types do, including mNbM (*Foxa2*, *Lmx1a*, and *Otx2*), mNbDA (*Foxa2*, *Lmx1a*, and *Wnt5a*), and mDA0 (*Foxa2*, *Lmx1a*, *Otx2*, and *Wnt5a*). Indeed, the only human post-mitotic cell type expressing more than one of these factors is hDA0 (*FOXA2* and *LMX1A*) (Figure 1C) (La Manno et al., 2016). These results suggest that TFs may be more difficult to detect in human tissue and/or that human post-mitotic cell types may not require the expression of as many or as high expression levels of early genes compared to their homologous mouse cell types. Future experiments should aim to distinguish between these possibilities.

### Factors Controlling mDA Neurogenesis

In the mouse, mDA neurogenesis is controlled by the pro-neural basic helix-loop-helix TF *Neurog2* (Andersson et al., 2006a; Kele et al., 2006). This TF is expressed in homologous human and mouse NProg and NbM (Figures 1A,B), indicating that neurogenesis occurs in the same cell type in both species. In agreement with this, other genes involved in neurogenesis are also expressed in these homologous cell types, such as *ASCL1* (in NProg), or *NEUROD1* (in NbM). Additionally, in both species, NProg cells share the expression of *SOX2* and *FOXA2* (Figure 1A), whereas NbM share the expression of *FOXA2*, *TUBB3*, and *DCX* (Figures 1C,D). Nevertheless, the expression of other TFs that regulate mDA neuron development differs greatly in the two homologous cell types. While the mouse NProg express several early genes such as *Lmx1a*, *Otx2*, *Rspo2*, *Msx1*, and *Corin* (Figure 1A), human NProg express later stage neuronal genes found in mNbM such as *NEUROD1*, *TUBB3*, and *DCX*, or even in mNbDA such as *DDC* (*DOPA decarboxylase*) (Figure 1). Similarly, mouse NbM express more early genes than human NbM, such as *Lmx1a*, *Otx2*, *Rspo2*, and *Ascl1* (Figure 1C). These results suggest that mouse NProg and NbM not only express earlier developmental genes, but they might be at an earlier developmental stage than human NProg and NbM. In agreement with this, our global transcriptomic analysis of the temporal differences between mouse and human NbM (Figure 2D) also suggests that mouse NbM cells are at an earlier homologous developmental time compared to human NbM.

### Factors Expressed in Post-mitotic mDA Cell Types

At first glance, the genes that define post-mitotic cell types in the mDA lineage are largely conserved in homologous cell types, but several differences can also be observed (Figures 1C,D). Expression of the nuclear receptor *Nr4a2* in post-mitotic neuroblasts is required for the acquisition and maintenance of the mDA phenotype (Zetterström et al., 1997; Castillo et al., 1998; Saucedo-Cardenas et al., 1998; Kadkhodaei et al., 2009). In the mouse, *Nr4a2* is expressed at high levels in NbM cells and is maintained in all cell types of the mDA lineage (NbDA; DA0-2), whereas human *NR4A2* is only expressed at high levels in embryonic mDA neurons (DA0-2), suggesting that differences in gene regulation exist between mouse and human. Additionally, immunocytochemical analysis of fetal human VM tissue has indicated that NR4A2 protein is present in cells that have not yet acquired the expression of *TH*, pointing toward the existence of human DA neuroblasts (NbDA) that express *NR4A2* (Villaescusa et al., 2016), which were not detected by scRNA-seq (La Manno et al., 2016). These differences in transcript and protein detection may be explained by differential regulation of transcription and translation as well as differences in the regulation of transcript and protein stability across species.

After neurogenesis, the first cell type of the mDA lineage found in the mouse is the mNbDA, a cell type with a transcriptional profile similar to that of hDA0 (Figure 1C,D). Indeed, both of these cell types express *Nr4a2* and additional TFs that regulate the differentiation and survival of mDA neurons, such as *Pbx1* (Villaescusa et al., 2016), *Pitx3*, and *En1* (Veenvliet et al., 2013). In addition, mNbDA cells express the vesicular monoamine transporter (*Slc18a2*), also found in mDA0, m/hDA1, and m/hDA2 (Figure 1D). However, mNbDA and hDA0 differ in that hDA0 cells express *TH*, whereas mNbDA cells do not.

The earliest post-mitotic cell types of the mDA lineage in mouse and human (mNbDA, mDA0, and hDA0), are relatively immature and do not express the dopamine transporter (*Slc6a3*) or Calbindin1 (*Calb1*), which in both species are found in the two mature embryonic mDA neurons, DA1 and DA2 (Figure 1D). Mouse and human DA1 neurons differ from the preceding DA0 neurons in their degree of maturation, as assessed by expression of *Slc18a2* (*Vmat2*) and *Slc6a3* (*Dat*). Additionally, DA2 neurons in both species express *ALDH1A*, an enzyme involved in the synthesis of retinoic acid (McCaffery and Drager, 1994), and *LMO3*, a TF highly enriched in the murine SNc, which functions as a transcriptional co-regulator of *PITX3* (Bifsha et al., 2017) and has also been suggested to play a role in Parkinson’s disease (Briggs et al., 2015).

All mouse and human DA neurons (except hDA0) share the expression of *BNC2*, a zinc finger TF primarily expressed in adult DA neurons of the SNc (Dougherty, 2017). This TF is known to regulate pigmentation of human keratinocytes (Jacobs et al., 2013) and GWAS studies have indicated that SNPs in this gene could be associated with Parkinson’s disease (Hook et al., 2018). On the other hand, a consistent difference between mouse and human DA neurons (DA0-2) (Figure 1D) was the expression of significantly higher levels of doublecortin (*DCX*) in human neurons than in mouse. Since Dcx is a microtubule associated protein essential for neuronal migration (Gleeson et al., 1999), this result may reflect that human DA neurons need to migrate for longer distances than their mouse counterparts. Other genes that were differentially expressed in a species-specific manner in DA neurons were *FOXA2*, which was expressed in human but not murine DA1 neurons, and *Lmo3*, expressed in mouse but not human DA0. Overall, the DA neuron subtype that exhibited the greatest species differences was DA0, with mouse DA0 expressing TFs related to DA development/function at higher levels (*Otx2*, *Wnt5a*, *Bnc2*, and *Lmo3*) than human DA0 (Figures 1C,D). However, it should be noted that a set of late TFs that regulate mDA neuron differentiation (*NR4A2*, *PBX1*, *PITX3*, and *EN1*) were found in all DA neuron subtypes (DA0-2) (Figure 1D), suggesting the existence of very robust and common late differentiation mechanism in both species.

In sum, while we found important species differences in the expression of early marker genes in proliferating cell types (Figure 1A), the expression of late marker genes in post-mitotic cell types varied less (Figure 1D). Furthermore, even within post-mitotic cells, gene expression in earlier homologous cell types, that is neuroblasts and DA0 neurons, varied more than in DA1-2, indicating that homologous differentiated cell types are more similar than homologous undifferentiated cell types. Finally, it is currently unclear which murine or human adult postnatal mDA neuron subtypes (Poulin et al., 2014, 2018; La Manno et al., 2016; Nichterwitz et al., 2016; Kee et al., 2017; Hook et al., 2018; Kramer et al., 2018; Zeisel et al., 2018; Tiklová et al., 2019a) are generated by each of the embryonic DA0-2 neurons and whether species differences may exist. Nevertheless, two lines of evidence suggest that embryonic DA2 neurons may be the precursors of SNc neurons in both species. First, the observation that DA2 neurons express several factors that are later found in the SNc, including *Sox6* (Panman et al., 2014), *ALDH1A1* (Galter et al., 2003), *Rgs6* (Bifsha et al., 2014), and *Lmo3* (Bifsha et al., 2017); and second, these genes have been associated to PD either by GWAS studies (*SOX6*, Dube et al., 2019), microarray data in PD post-mortem samples (*LMO3*, Briggs et al., 2015) or the presence of Parkinsonian symptoms in *Rgs6^–/–^* mice (Luo et al., 2019). Ultimately, lineage tracing experiments using either human embryonic tissue or hES cells differentiated into midbrain cell types will be necessary to define the lineage relationship between embryonic and postnatal/adult human mDA neuron subtypes.

Taken together, analysis of the developing mouse and human VM at the single-cell level indicates that while a common pool of TFs is necessary to generate mDA neurons in both species, their expression is not always found in homologous cell types. This may lead to the formation of species-specific signaling complexes and transcriptional networks and/or their activation for different lengths of time, which may result in distinct signaling strengths and biological outputs in different species. These are intriguing possibilities that require further investigation.

## Analysis of Dopaminergic Differentiation of Human Pluripotent Stem Cells

Advancements in the development of human stem cell-based cell replacement therapies for Parkinson’s disease have recently led to hPSC-derived products entering the clinic, with clinical trials either in preparation (Studer, 2017; Sonntag et al., 2018; Barker et al., 2019) or currently underway (Takahashi and Price-Evans, 2019). However, the composition and quality of such cell preparations have not been examined in detail at the single-cell level. Therefore, it is currently unknown how well hPSC-derived cells resemble the endogenous fetal VM cell types previously used for transplantation. We previously performed a comprehensive scRNA-seq analysis of developing human fetal VM tissue, dissected in the same way and from the same stages as used for transplantation in PD patients. This data has been used as a standard to assess, in an unbiased manner, the cellular and molecular composition of hPSC preparations differentiated into midbrain cell types (La Manno et al., 2016). We believe that such datasets combined with machine learning methodologies will routinely be used in the near future to evaluate both the composition and quality of *in vitro* derived dopaminergic cell preparations to be used for transplantation in PD patients. Furthermore, such methods can be used to develop and establish the standards of cell quality necessary to qualify cell preparations for transplantation. In this section, we exclusively focus on the cellular composition of hPSC-derived mDA preparations *in vitro*, from a single cell perspective. For additional information on cell replacement therapy, including cell transplantation strategies, functionality, and outcomes in PD models, the reader is directed to existing reviews (Allan et al., 2010; Arenas et al., 2015a; Barker et al., 2015, 2019; Lindvall, 2016; Björklund and Lindvall, 2017; Studer, 2017).

In our analysis of human stem cell differentiation into mDA neurons (La Manno et al., 2016), stem cell preparations were differentiated using the protocol developed by Kriks et al. (2011). Cells were analyzed at different stages of differentiation to establish a trajectory from the pluripotent stem cell state, through the intermediate progenitor and neuroblast states, until the generation of dopaminergic neurons. This strategy allowed us to assess whether cells differentiating *in vitro* recapitulate key stages and cell types found in human embryonic midbrain development. The fidelity of the cell preparations was first determined by directly comparing the transcriptomes of the cells in the preparation to that of the cell types found in the developing human midbrain. In addition, supervised machine learning was used to identify key features of the endogenous human VM cell prototypes and to evaluate the stem cell products based on the probability of an individual cell being any of the human VM cell types (La Manno et al., 2016).

Direct comparison of the transcriptomes of cells differentiating *in vitro* to that of cells found in the developing midbrain *in vivo* revealed that hPSC-derived cells appeared to share a varying range of transcriptional similarity with the *in vivo* standards, indicating that the cellular identity of hPSC-derived cells is not as well resolved (La Manno et al., 2016). However, the magnitude of these differences was similar to that observed between endogenous human and mouse midbrain cell types during development, indicating that the overall similarity or quality of hPSC-derived cells obtained by the Kriks protocol is good, but that there might be some differences compared to the endogenous standards.

### Quality Differences Between Endogenous and hPSC-Derived Cell Types

**FIGURE 3:**
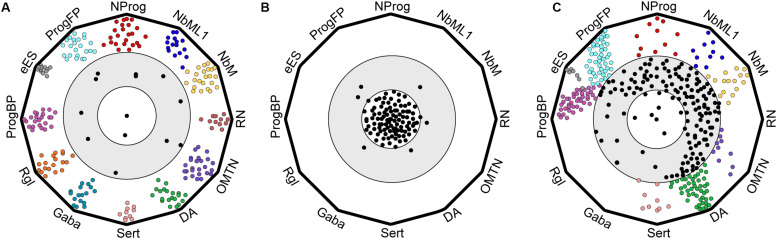
Cartoon of polygon plots showing differences in cell type composition and quality between endogenous ventral midbrain and hPSC-derived midbrain cell types. Cells were evaluated by machine learning (logistic regression) as described in La Manno et al. (2016). **(A)** Endogenous ventral midbrain (VM) cell types used in the training dataset show similarity to their prototypes. Each vertex of the polygon defines a prototype and cells are represented as circles. Most endogenous VM cell types are in the outer circle, close to their prototypes. **(B)** Negative control showing randomized gene expression in cell types. Random cells cluster far away from the standards, in an inner circle, that defines cells with no midbrain identity. **(C)** hESCs differentiated into midbrain cell types with a protocol based on Kriks et al. (2011) give rise to well-defined midbrain cell types that resemble their *in vivo* prototypes (cells in outer circle), but also to less defined midbrain-like cells (intermediate gray zone) that are clearly distinct from non-midbrain cells (inner circle).

A detailed comparison between hPSC-derived and endogenous VM cell types by machine learning (using logistic regression) revealed significant differences between these two cell preparations. While human endogenous cells were all recognized as very similar to their respective prototypes as defined by machine learning (Figure 3A) and random gene expression was readily detected (Figure 3B), hPSC-derived cell types were generally less well differentiated and exhibited more phenotypic variation (Figure 3C). However, some of the cell types derived from hPSCs resembled their *in vivo* counterparts more closely. For instance, hPSCs generated high-quality human floor plate progenitors (hProgFP, a standard combining hProgFPL and hProgFPM) and good quality basal plate progenitors (hProgBP). At the neuroblast stage, fewer cells acquire a well-characterized NbM and NbML1 identity, but a good number of high quality dopaminergic neurons (hDA, a standard combining endogenous hDA0-2) are generated (Figure 3C). hPSC-derived mDA neurons expressed multiple key mDA markers such as *NR4A2*, *TH*, and *PBX1*, but failed to express genes which commonly define hDA1-2, such as *DAT/SLC6A3* and *BNC2*, or that define a specific embryonic mDA subtype, such as hDA2 (*ALDH1A1*). These differences may reflect the fact that the protocol used for the differentiation of hPSCs into mDA neurons (Kriks et al., 2011) does not mimic all aspects of *in vivo* mDA neuron development.

### Differences in Cell Transitions Between Endogenous and hPSC-Derived Cell Types

Another feature that distinguished endogenous VM cell types from hPSC-derived cells in the machine learning analysis was the apparent difference in how differentiating cells transition from one intermediate cell type to another (La Manno et al., 2016). While each endogenous VM cell type was found to be very similar to its machine learning standard and cell types were clearly different from one another (Figure 3A), hPSC-derived cell types were less distinct and less well-defined along the differentiation axis; thus occupying an undefined gray zone in transcriptomic space (Figure 3C), which is distinct from cells in which the expression of midbrain genes was scrambled (Figure 3B) (La Manno et al., 2016). Since cells normally transition from one cell type to the next during embryonic development, e.g., from progenitor to neuroblast and finally to neuron, the clear separation between each endogenous cell type suggests that these transitions happen relatively fast *in vivo* and the probability of capturing them is rather low. Conversely, the presence of a continuum of cell types in hPSC-derived cultures suggests that such transitions may be much slower *in vitro*, as many cells appear to be captured while transitioning from one cell type to the next. Thus, it appears that endogenous midbrain progenitors differentiate following a sequence of events connecting well-defined and stable intermediate states through rapid transitions to generate the mature state. In contrast, cells differentiating from hPSCs *in vitro* go through a more constant differentiation flow characterized by less defined and less stable intermediate states. These differences in differentiation kinetics might be related to the lack of sufficient spatial information and the asynchronous nature of cells differentiated as a monolayer in two-dimensional (2D) cultures. This is in contrast with normal embryonic development, in which progenitor cells are immersed in a three-dimensional (3D) structure and surrounded by specific micro-environments, referred to as niches. These niches are composed of neighboring cells, which provide spatially arrayed information, such as mechanical forces and secreted factors, including morphogens, growth factors, extracellular matrix, neurotransmitters, and metabolites. It remains to be determined whether moving away from the traditional 2D culturing systems and toward 3D culturing systems may improve the molecular definition of individual cell types. Indeed, cells grown in 3D cultures tend to self-organize into discrete domains, and integrate more signals from neighboring cells (Kadoshima et al., 2013). In recent years, 3D culture systems have gained popularity and have been used to generate midbrain-like organoids containing functional dopaminergic neurons (Jo et al., 2016; Smits et al., 2019). Future experiments should thus aim to determine whether developmental transitions and overall cell quality can be improved by incorporating additional factors into the differentiation protocols and/or differentiating hPSCs as midbrain organoids.

### Differences in Cell Composition Between Endogenous and hPSC-Derived Cell Types: Therapeutic Implications

In recent years, improved protocols for the derivation of functional mDA neurons from stem cell preparations have led to renewed interest in cell transplantation as a viable treatment option for Parkinson’s disease. Nowadays, mDA neurons can be derived from hPSCs in a GMP-compliant manner (Doi et al., 2014; Kirkeby et al., 2017; Nolbrant et al., 2017). These efforts have recently culminated in the start of the first clinical trial involving hPSCs as a cell source for transplantation (Takahashi and Price-Evans, 2019). With the field moving quickly toward clinical applications, the ability to confidently assess the composition and quality of hPSC-derived mDA preparations has become increasingly important. However, few studies have examined the composition of hPSC-derived midbrain preparations at the single-cell level. In our study (La Manno et al., 2016), we used machine-learning analysis to assess our hPSC cultures differentiated toward an mDA fate using the Kriks protocol and found that these cells give rise to non-dopaminergic neurons, including serotonergic neurons and a few oculomotor and trochlear neurons (OMTN). However, other neuronal cell types such as red nucleus or GABAergic neurons were not identified (Figure 3C). More recently, Tiklová et al. (2019b) used scRNA-seq to analyze the general composition of hPSC derived midbrain cultures differentiated with a more recent protocol (Nolbrant et al., 2017) before and after transplantation in a rodent model of PD. Surprisingly, a large number of cells were identified as fibroblast-like vascular leptomeningeal cells (VLMCs), a cell type neither found in the developing human VM (La Manno et al., 2016), nor after transplantation of human fetal VM tissue in a rodent model of PD (Tiklová et al., 2019b). The functional impact of VLMCs on the rest of the transplanted cells and/or the host rodent brain remains to be explored. Similarly, the consequences of transplanting these cell types in a clinical setting are unknown since previous clinical trials were performed using human VM tissue, which does not contain VLMCs. Previous studies have suggested that the presence of non-midbrain cell types, such as serotonergic neurons, may underly the cause of graft-induced dyskinesias (Politis et al., 2010), arguing for the need to restrict cell transplantation to at least only those cell types present in the VM. It will therefore be of the utmost importance to determine the functional consequences of transplanting hPSC-derived preparations with and without VLMCs in animal models of PD prior to their application in cell replacement therapy for PD. We believe that a detailed analysis of the cellular and molecular composition of hPSC-derived preparations is necessary to progress toward a safe and efficient clinical application of stem cell-based cell replacement therapies for PD. In this context, we envision that scRNA-seq analysis will be increasingly used and required to assess the molecular composition of stem cell preparations and to determine whether the quality of cells in the preparation is comparable to that of the desired endogenous prototypes.

### How Can We Improve Current hPSCs Preparations for PD Cell Replacement Therapy?

Concerning cell composition and cell quality, there are a few remaining challenges and some opportunities for improvement. To date, the challenge remains to achieve cell preparations containing only midbrain cell types or better yet, only the midbrain cell types required for cell replacement. Regarding the tissue, it is clear that cell preparations should exclusively contain cells present within the VM, the tissue that has demonstrated therapeutic value and provided proof of principle for cell replacement therapy in PD. However, future cell replacement strategies would be expected to achieve cell preparations further enriched in the cell type/s that need to be replaced and thus hold the greatest therapeutic value, such as precursors of SNc neurons. At the same time, it would be desirable to reduce the midbrain cell types that do not need to be replaced and do not contribute to the development, maintenance, or integration of the transplanted cells. With increasing knowledge of the cell composition and function of individual cell types during development and in the adult brain, we foresee the next step as being the generation of designer cell preparations containing the cell types required and sufficient to achieve the desired therapeutic effect without adverse effects. In this scenario, scRNA-seq and other single-cell technologies (discussed below) will have an increasingly important role. For instance, scRNA-seq analysis can be used in an iterative process where the information gained from the analysis and comparison of endogenous cell types with hPSC-derived cell types is used to guide the improvement of differentiation protocols. For example, the presence of non-midbrain cell types in the culture, or differences in gene expression identified in midbrain cell types derived from hPSCs compared to their *in vivo* counterparts can highlight genes and pathways that are aberrantly expressed and/or regulated *in vitro*, resulting in a change in cell composition or quality. Once these differences are identified, such genes or pathways could be targeted for activation or inhibition, so that the differentiation of stem cells is optimized to resemble endogenous brain standards as closely as possible.

The next challenges for cell replacement therapy in PD with regard to cell composition and quality will then be two-fold. Firstly, subtype specification and late-stage maturation could be improved to generate cell preparations that are enriched in progenitor cells capable of selectively giving rise to mDA neurons of the ventral tier of the SNc. Secondly, those midbrain cell types unnecessary for transplantation should be identified and then eliminated from the culture so that only those cell types required for cell replacement would be transplanted. Additionally, future cell preparations could also be endowed with additional properties that may enhance their therapeutic value. For instance, by generating universal donor cells which cannot be targeted by the host immune system (Deuse et al., 2019), the need to immunosuppress the host would be eliminated. Another example would be the generation of cells resistant to the transmission of disease from the host brain to the transplanted cells, as has previously been described (Brundin et al., 2008); or the generation of cells resistant to disease by enhancing neuroprotective mechanisms and eliminating factors contributing to pathology. For such enhanced cells, we similarly believe that by systematically addressing specific pathways and molecular mechanisms at the single-cell level and by identifying which parameters are important for graft outcome, it may be possible to develop cell replacement therapy beyond the current state of the art and into the future.

## Future Opportunities

Recent advances in the field of single-cell biology are making it possible to gain more information of not only gene expression but also gene regulation and function at the single cell level. For instance, it is now possible to sequence a much greater number of cells per sample at increased depth and resolution, thus increasing the information gained from each precious human fetal sample (Svensson et al., 2018). At the same time, new computational tools such as RNA velocity and scVelo allow to infer lineage relationships from scRNA-seq data (La Manno et al., 2018; Bergen et al., 2019). At the same time, genetic lineage tracing has evolved from using exogenous barcodes (Kester and van Oudenaarden, 2018) to the use of endogenous somatic mutations in mtDNA combined with scRNA-seq or ATAC-seq (Ludwig et al., 2019; Xu et al., 2019). During the last years, multiple spatial transcriptomics methods have also been developed, ranging from multiplexed single-molecule fluorescent *in situ* hybridization (FISH) methods, such as osmFISH (Codeluppi et al., 2018), seqFISH (Eng et al., 2019), and MERFISH (Xia et al., 2019); to sequencing-based methods, such as *in situ* sequencing (ISS) (Ke et al., 2013; Lee et al., 2014), Slide-seq (Rodriques et al., 2019), STARmap (Wang et al., 2018), and high definition spatial transcriptomics (HDST) (Vickovic et al., 2019), which provide positional information of molecularly defined cell types in a tissue.

In addition, several other single-cell methods that provide information about the cellular state beyond the transcriptome have recently been developed. In the field of single-cell epigenomics, several methods have now been developed that can be used to shed light on the epigenetic landscape, thus making it possible to gain information about the gene regulatory networks that govern lineage commitment and cell fate decisions during development. Several methods have been developed that provide information about chromatin accessibility, e.g., single-cell ATAC sequencing (Buenrostro et al., 2015) and single-cell hypersensitive site sequencing (scTHS-seq) (Lake et al., 2018), whereas methods such as single-cell chromatin immunoprecipitation sequencing (scChIP-seq) (Rotem et al., 2015) and CUT&Tag (Kaya-Okur et al., 2019) can elucidate chromatin state through histone modifications. Moreover, methods such as single-cell combinatorial indexed Hi-C (Sci-Hi-C) (Ramani et al., 2020) and DamID (Kind et al., 2015) can provide information about chromosome configuration and genome organization. In the near future, other methods in the emerging fields of single-cell proteomics and metabolomics are expected to shed additional light on cell phenotype at the single-cell level (Ali et al., 2019; Yang et al., 2019), thus allowing for a more dynamic picture of cellular heterogeneity. Finally, any information gained from such single-cell analysis will need to be validated through functional genomic analysis. CRISPR based genetic screens can be used to validate gene targets identified through single-cell analysis, either at the individual gene level or through pooled CRISPR screenings. Recently, several methods that couple pooled CRISPR screens with a single-cell transcriptomic read-out have been developed (Dixit et al., 2016; Jaitin et al., 2016; Datlinger et al., 2017; Feldman et al., 2019), making it possible to elucidate complex gene regulatory networks in a heterogeneous cell population such as the developing VM.

## Conclusion

Until recently, most of our knowledge about mDA neuron development has come from studies in mice and little was known about the developmental programs that govern human mDA neuron development. Our scRNA-seq analysis of the developing human and murine VM has now generated a dataset that encompasses early mDA neuron development in both species, allowing us to study human development in an unbiased manner and to compare it to our current knowledge from the mouse (La Manno et al., 2016). From this analysis, we could identify several differences between human and murine VM development with regards to cell type composition, gene expression, and the temporal dynamics of VM development. Our results therefore highlight the need to use human midbrain development as the standard to tailor hPSCs-derived cells to the desired human prototypes, thus further improving the composition of cell preparations and the quality of the cells aimed for cell replacement in PD. It should also be noted that the single cell field is moving very rapidly into new technologies and it remains to be determined whether future scRNA-seq analysis of a greater number of cells from more samples at additional developmental time points and with greater sequencing depth may lead to the identification of additional cell types or subtypes, or to a better molecular definition of the cell types described in this review.

Future studies should aim to correlate the molecular signature of cell types identified by scRNA-seq in the mouse and human to their morphological properties (e.g., shape, size, dendrites and axonal projections), as well as their functional properties both *in vitro* and in animal models of PD (e.g., synaptic afferents and efferents, electrophysiological properties, neuronal activity and behavioral output). Moreover, by adopting a multi-omics approach and integrating different levels of information at the single-cell level, followed by functional genomics strategies for validation, it may be possible to understand and address the differences observed between the murine and human VM during development. Additionally, these same strategies could be used to identify differences between the healthy and Parkinsonian adult brain. Furthermore, these technologies will help us to identify and address any discrepancies between brain endogenous and stem cell-derived cell types, leading to the development of improved cell preparations and cell replacement therapy for the future treatment of PD.

## Author Contributions

All authors listed have made a substantial, direct and intellectual contribution to the work, and approved it for publication.

## Conflict of Interest

The authors declare that the research was conducted in the absence of any commercial or financial relationships that could be construed as a potential conflict of interest.
